# Clinical evaluation and nasoendoscopic scores of patients with chronic rhinosinusitis in Ilorin, Nigeria

**DOI:** 10.11604/pamj.2021.38.396.26115

**Published:** 2021-04-23

**Authors:** Samuel Oluyomi Ayodele, Olushola Abdulrhaman Afolabi, Segun Segun-Busari, Habeeb Kayodele Omokanye, Biodun Sulyman Alabi, Foluwasayo Emmanuel Ologe

**Affiliations:** 1 Department of Ear, Nose and Throat, University of Ilorin Teaching Hospital, Ilorin, Nigeria

**Keywords:** Chronic rhinosinusitis, nasal endoscopy, intranasal polyps, DIP score

## Abstract

**Introduction:**

chronic rhinosinusitis (CRS) is characterised by inflammation of the mucosal lining of the nose and paranasal sinuses for at least 12 weeks duration. Other than the diagnostic criteria that is based on clinical features; nasoendoscopy and/or computerized tomographic scan have been included in the diagnosis. This study seeks to outline the clinical evaluation and nasoendoscopic assessment of CRS patients.

**Methods:**

a hospital-based analytical study carried out on 154 participants. Clinical assessment and nasoendoscopy were performed and scored according to the discharge, inflammation and polyps/oedema (DIP) scale. Statistical analysis was carried out and results were presented in charts and tables.

**Results:**

of the 154 participants, 71 (46.1%) were males and 83 (53.9%) females with a male to female ratio of 1: 1.7. Nasal discharge and blockage were the commonest symptoms. Nasoendoscopy had higher yield in the examination of intranasal polyps (NPs) over anterior rhinoscopy. The prevalence of NPs was 26.6%. The result of DIP nasoendoscopic findings revealed more participants with moderate scores. There was a significant statistical difference between the presence of NPs on nasoendoscopy and DIP score.

**Conclusion:**

nasoendoscopy is an important aspect in the diagnosis and evaluation of patients with CRS. It provides a better visualization of NPs; therefore, it should be made routine in the clinical assessment and treatment of patients with CRS. The nasal endoscopic scoring of CRS should be considered as a common practice in clinical setting as well.

## Introduction

Rhinosinusitis (RS) is an inflammation of the mucosal lining of the nose and the paranasal sinuses characterised by two or more symptoms, one of which should be either nasal blockage/obstruction/congestion or nasal discharge (anterior/posterior nasal drip); with or without facial pain/pressure, reduction or loss of smell [1]. Rhinosinusitis is broadly classified into acute and chronic rhinosinusitis (CRS) based on the duration of the symptoms [1]. Chronic rhinosinusitis is therefore a group of disorders characterised by inflammation of the mucosal lining of the nose and paranasal sinuses for at least 12 consecutive weeks duration [1,2]. It is further classified into chronic rhinosinusitis with nasal polyps (CRSwNP) and chronic rhinosinusitis without nasal polyps (CRSsNP) [1,3]. Clinical diagnosis of CRS was made using the clinical practice guideline formulated by the rhinosinusitis task force of the American Academy of Otolaryngology, Head and Neck Surgery (AAO-HNS) and revised by the sinus and allergy health partnership (SAHP) [4]. The diagnostic criterion for CRS depends on clinical symptoms and signs which are categorised into minor and major criteria [2,5]. The classification relies on the identification of symptoms to make a diagnosis. The symptoms are divided into major symptoms nasal discharge (anterior/posterior) nasal congestion facial pressure or pain and hyposmia/anosmia and minor symptoms (headache, fever, halitosis, fatigue, dental pain, cough, ear pain, pressure, and/or fullness) [5]. When a patient presents with two major symptoms or one major and two minor symptoms for more than one hour on most days for 3 months or more [1,4,5]; then it is said to be diagnostic of [3]. Inflammation is documented by one or more of the following findings: purulent mucus or edema in the middle meatus or anterior ethmoid region, polyps in nasal cavity or the middle meatus, and/or radiographic imaging showing evidence of inflammation of the paranasal sinuses [4]. The nasal turbinates, which are erectile tissues in the lateral walls of the nasal cavities often respond to inflammatory changes and become engorged. Engorged nasal turbinates, oedematous nasal mucosa and mucopurulent anterior rhinorrhoea were the major signs found in a study by Sogebi *et al*. [6], and it is similar to the findings of da Lilly-Tariah in Port Harcourt, Nigeria [7]. Most of the clinical features of nasal obstruction, anterior and posterior nasal discharge, sneezing and facial congestion are common and make the diagnosis of chronic rhinosinusitis based on symptom criteria alone to be difficult [8,9]. Chronic diseases of the nose and the sinuses occur more often in adults and mostly involve the maxillary sinuses from where it can spread to involve other sinuses (multisinusitis). The spread is usually to the ethmoid, and frontal sinuses in that order, but rarely spread to the sphenoid sinus [6].

In the position paper on rhinosinusitis and nasal polyps by the European Academy of Allergy and Clinical Immunology, nasal endoscopy and/or computerized tomography (CT) scan findings have been included in the diagnosis [1]. Therefore, corroboration of the definitive diagnosis of CRS should be done with nasal endoscopy or CT scan [1]. Endoscopy provides adequate illumination and magnification of the nasal passages and is indicated for patients who experience chronic or recurrent acute rhinosinusitis symptoms, or those with suspected sinonasal polyposis [10]. This technique can be used more effectively than simple anterior rhinoscopic examination to diagnose anatomical variations that may cause nasal congestion, as well as to observe signs of crusting and pus indicative of ongoing infection [10]. In a comparative study, endoscopy detected nasal pathology in 38.7% more patients than anterior and posterior rhinoscopic examination [10,11]. Nasoendoscopy is a prerequisite for an accurate estimate of the prevalence of nasal polyps (NP), as not all patients that claim to have NP actually have polyps on nasal endoscopy [1]. During nasoendoscopy, the inferior nasal meatus, the middle nasal meatus with middle turbinate, the anterior wall of the sphenoid sinus, sphenoethmoidal recess and the superior nasal meatus with olfactory groove are inspected through the first, second and third passes [12,13]. Other endoscopic evaluations could include the size of the turbinates and examination of sinus ostia. The benefits of endoscopy are many, and the procedure is well tolerated by most patients. However, this procedure can result in mild pain or discomfort and occasional bleeding [14]. Existing endoscopy scales have limitations in complexity, validation, and/or applicability and there is an increasing need for a validated endoscopic grading system to assess rhinosinusitis severity [15]. Therefore, a novel and straightforward endoscopic scoring system measuring discharge, inflammation, and polyps/oedema (DIP) was invented [15]. It demonstrates a high correlation with existing scoring parameters like Lund Kennedy endoscopic score (LKES) and perioperative sinus endoscopy (POSE) [15]. The DIP scale is applicable to both the operated and unoperated patients. It is quick to complete, which is ideal for both clinical and research settings [15]. This study seeks to outline the subjective clinical evaluation and nasoendoscopic assessment of CRS patients attending ear, nose and throat (ENT) at University of Ilorin Teaching Hospital (UITH), Nigeria.

## Methods

A hospital-based analytical study conducted out at ENT department of UITH, Ilorin, Nigeria over a period of nine (9) months. Ethical approval for this study was obtained from the ethical review committee of the hospital before commencement. One hundred and fifty-four (154) consenting CRS patients aged 18 and above were recruited after a detailed explanation of the purpose of the study and procedures. Written informed consents were obtained. Clinical assessments which include history taking, ENT examination and nasal endoscopy was performed and tailored towards diagnosis and assessment of chronic rhinosinusitis. Diagnosis was made according to the clinical practice guideline formulated by the rhinosinusitis task force of the American Academy of Otolaryngology, Head and Neck Surgery (AAO-HNS) [2,5]. The endoscopic findings were recorded and scored with the discharge, inflammation and polyps/oedema (DIP) scoring system [15]. Each category was scored on an 11-point Likert scale from 0 to 10 based on the viewer´s overall assessment of the sinonasal mucosa; with a total of 30 points for each nasal cavity. Discharge, inflammation and polyp total score was classified into mild (0-10), moderate (11-20) and severe (21-30). For the discharge category: a score of 0 indicated absent discharge, 5 indicated thick mucus, and 10 indicated purulent discharge. For the inflammation category: 0 indicated no inflammation, 5 indicated moderate inflammation, and 10 indicated severe inflammation and for the polyp/edema category: a score of 0 indicated normal mucosa, 5 indicated marked edema/no polyps, and 10 indicated polyps filling nasal cavity [15,16]. Statistical analysis was carried out using SPSS (Statistical Product and Service Solutions) version 20. Results were presented in simple tables and figures. Descriptive statistics (mean, standard deviations and frequencies) were calculated for all measures. The association between continuous variables and specific outcome variables were tested with chi square test. For statistical studies, p value <0.05 was considered as being statistically significant.

## Results

**Figure 1:**
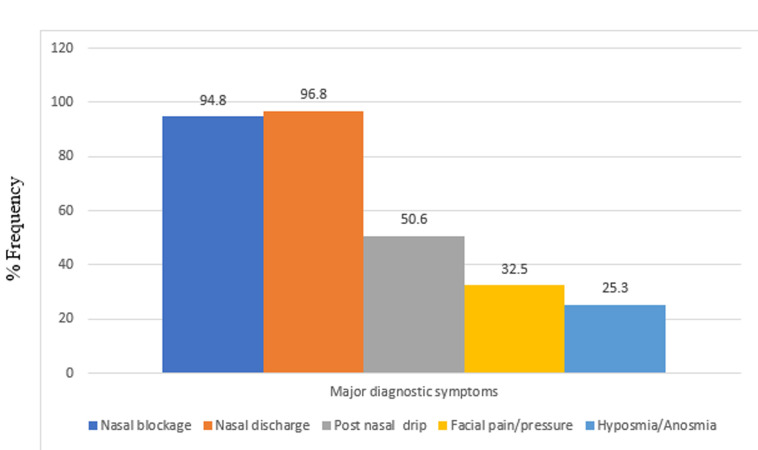
distribution of the major diagnostic symptoms of chronic rhinosinusitis

**Figure 2:**
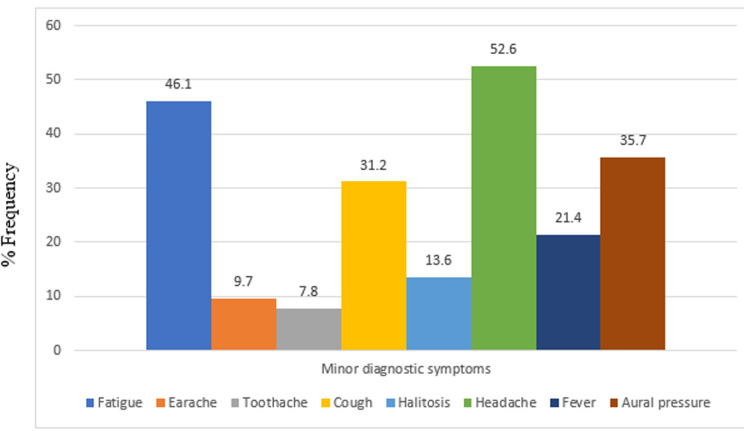
distribution of the minor diagnostic symptoms of chronic rhinosinusitis

This study was carried out on 154 patients with chronic rhinosinusitis recruited from ENT clinic within the study period. The age and sex distribution shown on Table 1 revealed the age range of participants was between 18 and 88 years with a mean age of 51.21 years ± 16.81. The highest number of participants 38 (24.7%) was found in age group 51-60 closely followed by 35 (22.7) participants in age group 61-70. The participants comprised of 83 (53.9%) females and 71 (46.1%) males with a male to female ratio of 1: 1.7. CRS ranged from 3 months to 20 years with mean duration of 3 years, 7 months, 3 week and 3 days (Mean = 43.88 ± 38.19). According to the distribution in Table 1, 74 (48.1%) participants has had CRS symptoms for 3 to 24 months at the time of study while 43 (27.9%) has had symptoms for 25 to 48 months. Figure 1 and Figure 2 outlined the major and minor diagnostic symptoms of CRS. Nasal discharge of at least 3 months duration was found in 96.8% of participants. This was closely followed by nasal blockage in 94.8%; then, postnasal drips, facial pain/pressure and hyposmia/anosmia with 50.6%, 32.5% and 25.3% respectively. Among those who presented with nasal discharge, participants with mucopurulent nasal discharge were found to be the commonest with 74 (48.1%) followed by those who presented with mucoid, 45 (29.2%) and watery, 23 (14.9%). Others, 12 (7.8%) had both mucoid and watery discharges alternating. This study also revealed that participants who complained of allergic symptoms were 88 (57.1%). Majority of the participants, 81 (52.6%) had partial nasal patency. Nasal discharge and engorged nasal turbinates were the commonest findings in the nasal cavities with over 70% of participants. Polyps were found in the nasal cavities of about 11.7% (Table 2). Among 41 participants who were found with intranasal polyps on nasoendoscopy, only 18 had intranasal polyps on anterior rhinoscopy (Table 3). There was a statistically significant difference in the relationship between the frequency of NPs found on anterior rhinoscopy and that found on nasoendoscopy (p value <0.001). Among the participants, 68 (44.2%) had moderate DIP score while 45 (29.2%) and 41 (26.6%) had mild and severe DIP score respectively (Table 3). Among the participants who had CRSwNP, 78% were found to have severe DIP score. The difference between the presence/absence of NPs and DIP score was statistically significant (p values< 0.001).

**Table 1:** age/sex distribution and duration of symptoms of chronic rhinosinusitis

Variables	Participants n=154	Percentage (%)
**Age groups**		
18 - 30	27	17.5
31 - 40	16	10.4
41 - 50	23	14.9
51 - 60	38	24.7
61 - 70	35	22.7
71 - 80	10	6.5
81 - 90	5	3.2
Mean ± SD	51.2 ± 16.8	
Range	18 - 88	
**Gender**		
Male	71	46.1
Female	83	53.9
**Duration of symptoms (months)**		
3 - 24	74	48.1
25 - 48	43	27.9
49 - 72	21	13.6
Above 72	16	10.4
Mean ± SD	43.9 ± 38.2	
Range	3 - 240	

**Table 2:** findings on anterior and posterior rhinoscopy

Variables	Participants	Percentage (%)
**Nasal patency** Good patency Partial patency No patency **Nasal cavity/mucosa**	47 81 26	30.5 52.6 16.9
Normal	31	20.1
Discharge	111	72.1
Polyps	18	11.7
Septal anomaly	10	6.5
**Nasal turbinates**		
Normal	13	8.4
Engorged	112	72.7
Pale/ bluish tinge	25	16.2
Hyperaemic	5	3.2

**Table 3:** chronic rhinosinusitis with and without nasal polyps and DIP Score

Variables	CRSwNP (%)	CRSsNP (%)	Total (%)	χ2	p
Anterior rhinoscopy	18 (11.7)	136 (88.3)	154 (100)		
Nasoendoscopy	41 (26.6)	113 (73.4)	154 (100)	11.091	<0.001*
**DIP Score**	**CRSwNP (%) n=41**	**CRSsNP (%)n = 113**	**Total (%) n = 154**		
Mild	1 (2.5)		45 (29.2)		
Moderate	8 (19.5)	44 (38.9)	68 (44.2)		
Severe	32 (78.0)	60 (53.1) 9 (22.0)	41 (26.6)	76.904	<0.001*

## Discussion

This study found a slightly high female preponderance which was similar to the findings of Sogebi *et al*. [6], da Lilly-Tariah [7], and Onotai *et al*. [17]. This could be because women are more concerned about their health and thus seek medical attention more promptly and often than men [18]. However, Fasunla *et al*. [19] and Afolabi *et al*. [20] found a slight male preponderance in their studies on patients presenting with rhinosinusitis. The mean duration of CRS symptoms revealed by this study was also within the range reported by previous studies where mean duration of CRS ranged between 2 to 6 years [6,7,21,22]. This high mean duration may be as a result of the fact that many of our patients in this environment tend to take the nasal symptoms as a common but simple problem. They therefore depend on self-medications until when the disease becomes persistent or manifest with other ominous sequels. Similarly, most patients would have visited other peripheral health centres before presenting to a tertiary institution with specialist clinics like ours. Nasal discharge and nasal blockage were common to almost all patients in this study. These symptoms were also accompanied by post nasal drip, hyposmia/anosmia and facial pain/pressure. These were in accord with the findings of other studies carried out in Nigeria [6,7,17,20,23,24]. Mucopurulent nasal discharge was found to be the commonest followed by mucoid and watery nasal discharge respectively. This implies that infective type of CRS is not uncommon in this environment. However, Sogebi *et al*. [6] in the South-West Nigeria found mucoid nasal discharge to be the commonest. In a two-year prospective study on the pattern of clinical features of chronic simple rhinosinusitis in Port Harcourt, da Lilly-Tariah [7] reported 72.7% cases of chronic infective rhinosinusitis as the commonest type of rhinosinusitis followed by vasomotor rhinitis with allergic rhinosinusitis being the least in his series. These were not consistent with other studies [6,17,20,24,25] where allergic rhinosinusitis was commoner when compared to infective CRS. In some of these studies [6,20,24], features of both acute and chronic types were considered together irrespective of the duration of the symptoms and allergy was found commonly among patients with acute type of rhinosinusitis. It is important to bear in mind that the predominant type of rhinosinusitis (in terms of cause) may vary depending on various environmental factors. In this study however, CRS patients who complained of allergic symptoms like excessive sneezing and nasal itching were about 57%. Also, in the study on the clinical profile and management of chronic rhinosinusitis among adults in North-Western Nigeria; allergy was seen to be the cause of CRS in 64.4% of patients [25]. The role of allergy in chronic rhinosinusitis has been debated for many years. It has been postulated that inflammation and swelling of the nasal mucosa in patients with allergic rhinitis can obstruct the sinus ostia, reduce ventilation, leading to mucus retention, and then predisposing them to developing CRS [1,26]. A document released by the joint task force on practice parameters (JTFPP) recommended that patients with recurrent RS or CRS should be evaluated for underlying allergy [27]. Similarly, Afolabi *et al*. pointed the fact that as many as 60% of patients with CRS have substantial allergic sensitivities [20]. Iseh and Makusidi [23] also attested that allergy remains a significant cause of chronic rhinosinusitis in this environment after infective causes.

Reduced nasal patency with nasal discharge and engorged nasal turbinates were the commonest findings in the nasal cavities in about 70% of CRS patients during anterior/posterior rhinoscopy this was followed by pale/bluish turbinates and presence of intranasal polypoid growths respectively. Similarly, engorged nasal turbinates, oedematous nasal mucosa and mucopurulent anterior rhinorrhoea were the major signs found in the study by Sogebi *et al*. [6] which is concurrent with studies of da Lilly-Tariah [7], Adegbiji *et al*. [24] and Mainasara *et al*. [25]. Al-Hassani *et al*. [28] found nasal obstruction from engorged turbinates the commonest feature in his work. The nasal turbinates are erectile tissues in the lateral walls of the nasal cavities, often respond to inflammatory changes and can become permanently engorged after repeated assaults. Most of the clinical features of nasal obstruction, anterior and posterior nasal discharge, sneezing and facial congestion are common and can also be found in some other nasal pathologies making the diagnosis of chronic rhinosinusitis difficult if based on symptom criteria alone [6]. Durr *et al*. [15] found that nasal endoscopy is an important aspect in both the initial evaluation, making diagnosis as well as follow up of patients with CRS. They also used the DIP scoring system for the nasal endoscopic examination which has the ability to provide an overall score for each nasal cavity. The result of nasoendoscopic findings (using the DIP score) in this study revealed more participants with moderate score. Among those who had CRSwNP, 78% was found to have severe DIP score. The correlation between the presence/absence of intranasal polyps and severity of DIP score was statistically significant (p value < 0.001). Among the 41 participants who were found with NPs on nasoendoscopy in this study, only 18 participants were detected with NPs on rhinoscopy. The correlation between NPs found on anterior rhinoscopy and on nasoendoscopy was also statistically significant. This confirms the superiority of nasoendoscopy over rhinoscopy with nasal speculum in the assessment of chronic rhinosinusitis; and supports the current trend of reliance on nasoendoscopy and/or CT scan to make a diagnosis of CRS. The prevalence of chronic rhinosinusitis with nasal polyps in this study (26.6%) was consistent with previous studies [22,24,29]. However, a systemic review and meta-analysis of chronic rhinosinusitis patients with nasal polyposis reported a low prevalence of chronic rhinosinusitis with nasal polyps ranging from 2.7% to 4.3% across Europe and the United States [30]. A higher prevalence was found by Durr *et al*. [15] where majority of patients (62.1%) had CRSwNP in their study, while Vaid *et al*. [31] reported CRSwNP in 50% of cases. Several other studies have found the addition of endoscopic findings to symptom-based criteria significantly improved the diagnostic accuracy of CRS and is superior to subjective symptom scores at detecting early pathological changes in patients with CRS [15,32,33]. Nasal endoscopy provides excellent visualization of polyps, especially small polyps. It also shows nasal polyps originating from contact areas in middle meatus [34].

## Conclusion

This study revealed that the presence of NPs confers severe endoscopic scores in CRS patients. Therefore, nasoendoscopy should be made routine in the clinical assessment and treatment of patients with CRS. We recommend that nasal endoscopic scoring of patients with CRS should be considered as a common practice in ENT clinical setting. Data obtained from these measures should be used in evaluating treatment effectiveness and can thus influence improvement in universal CRS management.

### What is known about this topic

- Nasoendoscopy detected nasal pathology in more patients than anterior and posterior rhinoscopic examination;
- Nasoendoscopy is a prerequisite for an accurate estimate of the prevalence of nasal polyps.

### What this study adds

- The result of nasoendoscopic assessment with DIP score in this study revealed more participants with moderate score among CRS patients in Nigeria;
- The correlation between intranasal polyps found on anterior rhinoscopy and on nasoendoscopy was statistically significant;
- Among the participants who had CRSwNP, 78% were found to have severe DIP score and the difference between the presence/absence of intranasal polyps and DIP score was statistically significant (p values < 0.001).
